# The synergistic effect of high temperature and ozone on the number of deaths from circulatory system diseases in Shijiazhuang, China

**DOI:** 10.3389/fpubh.2023.1266643

**Published:** 2023-10-03

**Authors:** Guiqin Fu, Haimin Cheng, Qian Lu, Huayue Liu, Xiaohui Zhang, Xingshan Zhang

**Affiliations:** ^1^China Meteorological Administration Xiong’an Atmospheric Boundary Layer Laboratory, Xiong’anChina; ^2^Key Laboratory of Meteorology and Ecological Environment of Hebei Province, Shijiazhuang, China; ^3^Hebei Meteorological Service Center, Shijiazhuang, China; ^4^Chengde Meteorological Service of Hebei Province, Chengde, China; ^5^Handan Meteorological Service of Hebei Province, Handan, China

**Keywords:** high temperature and ozone concentration, circulatory diseases, the number of deaths, synergistic effect, health effect

## Abstract

**Introduction:**

Urban ozone pollution in China is becoming increasingly serious. Climate warming, high temperatures, and ozone pollution all have significant impacts on human health. However, the synergistic effects of high temperatures and ozone pollution in summer on human health are rarely studied. China is at a critical stage of environmental pollution control. Assessing the health impact of high temperatures and ozone exposure on the number of deaths from circulatory diseases is of great significance for formulating ozone-related prevention and control policies.

**Methods:**

This study uses daily data on deaths from circulatory system diseases in Shijiazhuang from June to August during the summer of 2013–2016, as well as concurrent meteorological data and concentration of O_3_ and PM_2.5_ pollution data. The generalized additive model (GAM) with Poisson distribution, smooth curve threshold effect, and saturation effect method is used to control for confounding effects.

**Results:**

The study evaluates the impact of short-term exposure to temperature and ozone on deaths from circulatory system diseases and the synergistic effect after controlling for confounding factors. The results show that the impact of temperature and ozone on deaths from circulatory system diseases in Shijiazhuang is nonlinear, with a temperature threshold of 27.5°C and an ozone concentration threshold of 100 μg/m^3^. With an increase of temperature by 1°C, the risk of deaths for total population, men and women are 6.8%, 4.6% and 9.3%, respectively. The increase in temperature and ozone concentration has a greater impact on women; in men, the increase has a lag effect of 2 to 3 days, but the lag did not affect women.

**Discussion:**

In conclusion, high temperatures and high ozone concentration have synergistic enhancement effects on circulatory system diseases. Prevention and scientific management strategies of circulatory system diseases in high temperatures and high ozone environments should be strengthened.

## 1. Introduction

China used to suffer from serious air pollution. With the Chinese government’s efforts to reduce emissions, PM_2.5_ and other fine particulate matter pollution has been significantly improved. However, ozone (O_3_) pollution has become increasingly serious in recent years, and has become a serious problem for China’s urban environment (1–3). Especially against the background of global warming, the frequency and intensity of heat wave events has increased. The dual effects of high temperatures and ozone pollution may coexist for a long time, and will seriously threaten people’s health and become a new focus of attention (4–6).

According to the Lancet Countdown China report, the number of heatwave exposure days *per capita* in China increased by 4.51 days in 2020 compared to the 1986–2005 average, resulting in an increase of about 92% in heatwave related deaths, and the impact on human health is increasing. High ozone pollution can lead to an increase in related diseases and deaths (7–9). Previous studies focused more on the impact of one atmospheric environmental condition on health, such as high temperatures or ozone. However, there are few studies on whether high temperatures and ozone exposure have synergistic effects on human health.

The Beijing-Tianjin-Hebei region is an area with serious air pollution, and the ozone concentration has been on the rise in recent years (10, 11). Shijiazhuang, the capital city of Hebei Province, is also a representative city in northern China. In this paper, ozone concentration data published on the website of the Ministry of Environmental Protection of China during 2013–2016 and circulatory system disease deaths in Shijiazhuang during the same period are used. Based on epidemiological analysis, a generalized additive model and nonparametric binary response model are used to evaluate the effects of air temperature and short-term exposure to ozone on the number of deaths from circulatory system diseases in Shijiazhuang. The objective is to further explore the impact risks of high temperatures and O_3_ pollution on human health in an environment with multiple exposures, so as to strengthen the proactive prevention awareness of highly sensitive people and provide a basis for the government to formulate scientific prevention and control policies.

## 2. Data and methods

### 2.1. Data sources

This study is conducted in Shijiazhuang (114° 48’e, 38° 03’n), the capital of Hebei Province in northern China. The number of daily circulatory disease deaths in Shijiazhuang during the summer (from 1 June to 31 August) from 2013 to 2016 is obtained from the Chinese Center for Disease Control and Prevention. According to the 10th edition of the International Classification of Diseases (IDC-10, coded as I00-99), deaths from coronary heart disease, ischemic heart disease, ischemic stroke, cerebral hemorrhage, and cerebral infarction are included.

The data of daily mean temperature, relative humidity, and air pressure in Shijiazhuang during the summer (from 1 June to 31 August) from 2013 to 2016 are provided by Hebei Meteorological Information Center. Data of O_3_ (O_3_-8h) and PM_2.5_ average daily concentration of atmospheric pollutants during the same period are obtained from the website of the Ministry of Environmental Protection of China. All of this data are quality-controlled before being released by professional organizations.

### 2.2. Research methods

The daily death number of circulatory system diseases is calculated according to time series, and the influence of temperature and O_3_ short-term exposure on death number of circulatory system diseases is evaluated using a generalized additive model (GAM) of Poisson distribution (12, 13). Before assessing the synergistic effect of air temperature and O_3_ on the daily death number of circulatory system diseases, the influences of daily mean air temperature and O_3_ concentration on daily death number of circulatory system diseases are studied, respectively, to determine whether there is a curve relationship. In this model, the daily death number of circulatory system diseases is taken as the dependent variable, the air temperature (O_3_) as the independent variable, and the regression spline function is used to control the confounding effects of time trend (Time), annual change (Year), Holiday effect (Holiday), relative humidity (RH), and PM_2.5_ concentration. The partial autocorrelation function (PACF) is used to select the degrees of freedom for the time trend until the absolute value of the sum of PACFs reaches a minimum. The research formula is as follows:


$$\begin{array}{c} \log\left[Ε\left(Y_{t}|Χ\right)\right]=\alpha +\beta Χ+s\;\left(\mathrm{time},df=4\right)+s\left(PM_{2.5},df=2\right)\\ \;+RH+\mathrm{Year}+\mathrm{Holiday} \end{array}$$

In the formula, Y*_t_* is the daily death number of circulatory diseases on day *t*, E (Y*t*|X) is the expected daily number of deaths from circulatory system diseases on day *t*, α is the intercept, β is the regression coefficient, Χ is the temperature T (ozone O_3_), *s*() is a nonlinear spline function, and df is the degree of freedom.

Secondly, stratified thresholds of daily mean temperature and O_3_ concentration are analyzed according to threshold effect and saturation effect of smooth curves, and tested by logarithmic likelihood ratio. Third, the synergistic effect of air temperature and O_3_ on the daily death number of circulatory diseases is analyzed.

All results are expressed as relative risk (RR) and 95% confidence interval (95% CI), with *p* < 0.05 as the test of statistical significance. In addition, the impact on different genders is assessed.

## 3. Results

### 3.1. Background analysis of air pollution in Shijiazhuang

**Figure 1:**
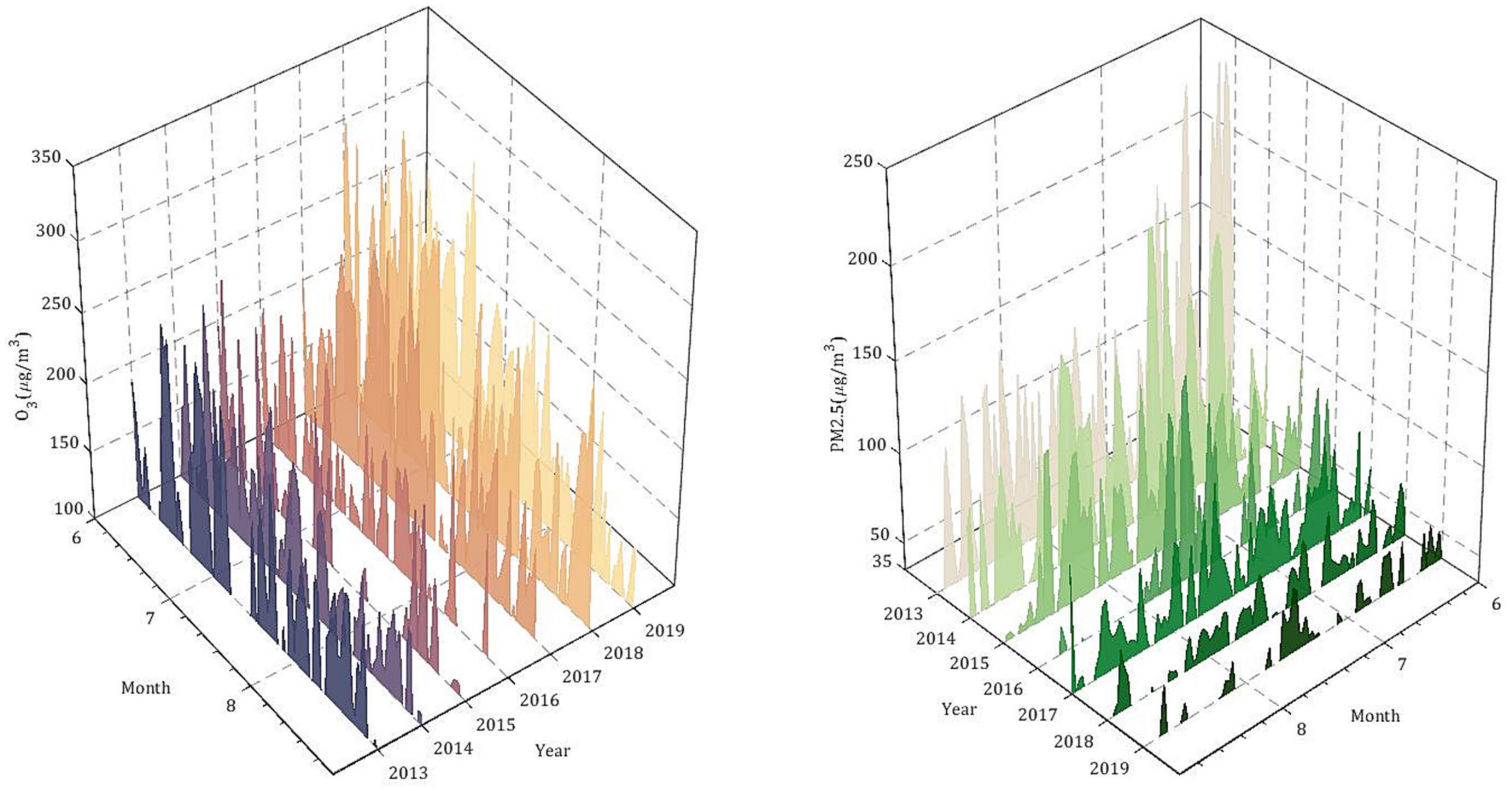
PM_2.5_ and ozone concentrations in summer (June to August) from 2013 to 2019 in Shijiazhuang City.

According to GB 9137–88 and HJ 633–2012, the concentrations of major air pollutants O_3_ and PM_2.5_ in Shijiazhuang are calculated from June to August in the summers of 2013–2019, and their concentrations relative to O_3_ < 100 μg/m^3^ (Grade I: excellent) and the number of days exceeding the standard compared with PM_2.5_ < 35 μg/m^3^ (Grade I: excellent) to analyze the change characteristics of air pollution in Shijiazhuang. Figure 1 shows the number of days when the concentration of main air pollutants O_3_ and PM_2.5_ exceeded the standard in Shijiazhuang and their variation trends. Over the past 7 years, as the government has stepped up to address pollution, the PM_2.5_ concentration in Shijiazhuang has dropped significantly in summer, with the number of days with PM_2.5_ exceeding 35 μg/m^3^ decreasing by 5.3 days per year on average. The average PM_2.5_ concentration in the summer of 2013 was 94.3 μg/m^3^, and by the same period in 2019 it was 34.3 μg/m^3^, which means the average PM_2.5_ air quality level throughout the summer is excellent. However, the O_3_ concentration shows an obvious upward trend. In the summer of 2013, the average O_3_ concentration was 137.3 μg/m^3^, while in the summer of 2019, the average O_3_ concentration was 137.3 μg/m^3^. The average O_3_ concentration increased by 27.6% over the past 7 years. The number of days exceeding the O_3_ standard increased from 66 days in 2013 to 84 days in 2019, with an annual growth rate of 3.6. It can be seen that ozone pollution should become a new focus of attention.

### 3.2. Statistical characteristics of circulatory system disease deaths, pollutant concentration, and meteorological elements in Shijiazhuang

Table 1 shows the statistical characteristics of circulation system deaths and meteorological environment elements in Shijiazhuang. In the summer (1 June to 31 August) from 2013 to 2016, 4,420 people died from circulatory diseases in Shijiazhuang city, of which 54.3% were men and 45.7% were women. The average daily death number from circulatory system diseases in Shijiazhuang city was 12.0, and the maximum daily death number was 40.0. During the corresponding period, the average daily temperature was 26.8°C, the relative humidity was 64.7%, O_3_ concentration was 123.6 μg/m^3^, and PM_2.5_ concentration was 71 μg/m^3^.

**Table 1:** Statistical characteristics of circulatory system disease deaths and meteorological elements and air pollutants in Shijiazhuang from June to August, 2013 to 2016.

		Average	Standard deviation	Min	P25	P50	P75	Max
Deaths from circulatory diseases	Total number	12.0	4.4	4.0	9.0	12.0	14.2	40.0
	male	6.5	2.9	0.0	4.0	6.0	8.2	21.0
	female	5.5	2.7	1.0	4.0	5.0	7.0	19.0
Daily mean of meteorological elements	T	26.8	2.6	19.7	25.1	27.0	28.6	33.1
	RH	64.7	15.8	24.0	53.0	65.0	77.0	99.0
	P	993.3	3.8	981.8	990.6	993.5	996.0	1004.7
Daily mean of air pollution	O_3_-8h	123.6	48.5	24.0	86.0	118.0	159.4	251.4
	PM_2.5_	71.0	44.8	3.0	36.0	62.8	95.0	241.1

### 3.3. Exposure-response relationship between daily mean temperature, ozone, and deaths from circulatory diseases in Shijiazhuang

**Figure 2:**
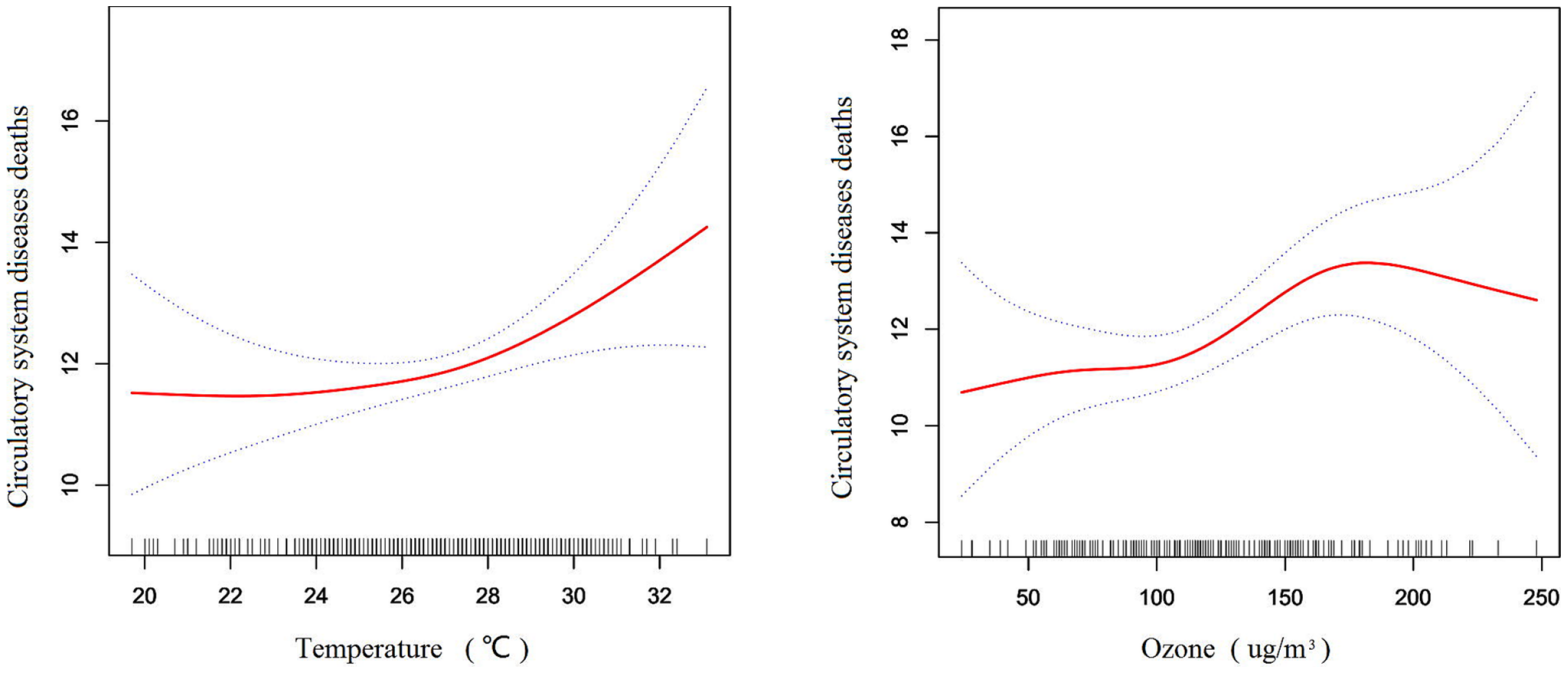
Variation curves of average temperature, O_3,_ and circulatory system disease deaths in Shijiazhuang from June to August, 2013 to 2016.

Figure 2 shows the exposure-response relationship between daily mean temperature, ozone concentration, and the number of deaths from circulatory diseases in Shijiazhuang. The model controls the confounding effects of time trend, annual change, holiday effect, relative humidity, and PM_2.5_ concentration. The daily mean temperature, ozone concentration, and the number of deaths from circulatory diseases show nonlinear changes. The increased risk of daily deaths from circulatory diseases is 1.6% (95%CI: 1.001, 1.032) for every 1°C increase in daily mean temperature. The increased risk of daily death from circulatory disease is 2.1% for every 10 μg/m^3^ increase in O_3_ concentration (95%CI: 1.007, 1.034). It can be seen that rising temperatures and increasing ozone concentration are associated with an increased risk of death from circulatory diseases.

### 3.4. Effects of air temperature and ozone on circulatory disease deaths under different thresholds

Based on the previous analysis, the smoothing curve threshold effect and saturation effect method are used to analyze the threshold effect of temperature and ozone on the death number of circulatory system diseases. Table 2 shows the relative risk and 95% confidence interval (95%CI) of the influence of daily mean temperature on the number of deaths from circulatory diseases under the stratification of temperature and ozone threshold. When the daily mean temperature is higher than 27.5°C, the daily death number of circulatory diseases changes steadily with temperature. The relative risk (RR) of increased daily deaths from circulatory diseases is 0.997 (95%CI:0.976, 1.019) for every 1°C increase in mean temperature. If the RR is less than 1, no risk relationship is found. The RR of daily deaths from circulatory diseases increases by 1.049 (95%CI: 1.017, 1.083) for every 1°C increase in mean temperature above 27.5°C. RR greater than 1 is associated with an increased risk of 4.9% and is tested for significance at *p* < 0.05. The log-likelihood ratio of air temperature between the two layers is 0.023, and the threshold stratification is statistically significant.

**Table 2:** Relative risk and 95% confidence interval (95%CI) for the effect of temperature and O3 on the number of circulatory system deaths in Shijiazhuang from June to August, 2013 to 2016.

Environmental element	Threshold value	RR (95%CI)	*p*
*T* (°C)	*T* < 27.5	0.997 (0.976, 1.019)	0.023
	*T* ≥ 27.5	1.049 (1.017, 1.083)*	
O_3_ (μg/m^3^)	O_3_ < 100	0.994 (0.965, 1.025)	0.063
	O_3_ ≥ 100	1.037 (1.015, 1.059)*	

*p < 0.05.

Similarly, when the O_3_ concentration is lower than 100 μg/m^3^, the RR for increasing the number of daily deaths from circulatory diseases is 0.994 (95%CI: 0.965, 1.025) with an increase of 10 μg/m^3^, and no risk relationship is found. When O_3_ concentration is higher than 100 μg/m^3^, the RR of daily deaths from circulatory diseases increases by 1.037 (95%CI: 1.015, 1.059) and the risk increases by 3.7% for every 10 μg/m^3^ increase in O_3_ concentration (*p* < 0.0001). The risk relationship passes the significance test.

### 3.5. Synergistic effects of air temperature and ozone on circulatory disease deaths

According to the stratification of ozone concentration and daily mean temperature threshold, the influence of every 1°C increase in daily mean temperature on the number of deaths from circulatory system diseases and the relationship between men and women are calculated, respectively, under different ozone concentrations and different temperature threshold intervals (Table 3). When the O_3_ concentration is less than 100 μg/m^3^, the temperature effects of different stratifications are different. When the temperature is less than 27.5°C, no significant risk relationship is found among the total population or among men. When the temperature is higher than 27.5°C, with an increase of temperature by 1°C, the risk of the total number of deaths from circulatory diseases is 6.8% (95% CI:0.929, 1.228), but there are only 33 samples, and *p* < 0.05 is not statistically significant. When the O_3_ concentration is greater than 100 μg/m^3^ and the temperature is lower than 27.5°C, no significant risk is found in the total population or among men. However, when the temperature is greater than or equal to 27.5°C, every 1°C increase in daily mean temperature has an increased risk for the total number of deaths from circulatory diseases, for men and women, of 6.8% (95%CI: 1.025, 1.114), 4.6% (95%CI: 0.988, 1.109), and 9.3% (95%CI: 1.029, 1.162). In conclusion, when the O_3_ concentration is greater than 100 μg/m3 and the air temperature is greater than 27.5°C, both the total number and the number of male and female deaths show the greatest risk effect value, indicating that higher air temperature and high ozone pollution have a synergistic enhancement effect on the number of circulatory deaths, especially for women.

**Table 3:** Relative risk and 95% confidence interval (95%CI) of circulatory system deaths with every 1°C increase between different concentrations of temperature and O_3_ interval.

People	O_3_ (μg/m^3^)	*T* (°C)	sample	Mean value	RR (95%CI)
Total number	<100	<27.5	97	11.8	0.992 (0.957, 1.027)
		≥27.5	33	13.1	1.068 (0.929, 1.228)
	≥100	<27.5	104	11.7	0.995 (0.955, 1.037)
		≥27.5	134	12.2	1.068 (1.025, 1.114)*
Male	<100	<27.5	97	6.4	0.969 (0.924, 1.017)
		≥27.5	33	6.9	1.029 (0.848, 1.247)
	≥100	<27.5	104	6.5	0.987 (0.934, 1.042)
		≥27.5	134	6.5	**1.046 (0.988, 1.109)**
Female	<100	<27.5	97	5.4	1.020 (0.968, 1.074)
		≥27.5	33	6.1	1.005 (0.944, 1.070)
	≥100	<27.5	104	5.1	1.115 (0.909, 1.368)
		≥27.5	134	5.7	1.093 (1.029, 1.162)*

*p < 0.05.

### 3.6. Lagged effects of temperature and ozone on deaths from circulatory diseases

Based on the analysis of the synergistic effect of temperature and ozone on the death number of circulatory system diseases, the lag effect is further analyzed. Table 4 shows the relative risk and 95% confidence interval (95%CI) of the total number of deaths in the circulatory system in Shijiazhuang for every 1°C increase in temperature when O_3_ ≥ 100 μg/m^3^ and T ≥ 27.5°C, with 0 to 9 days lag. It can be seen that the lag effect of the synergistic effect of temperature and ozone is more complex. The risk of 3 days lag (T.3) is 6.9% (95%CI:1.029, 1.111), and the risk of 9 days lag (T.9) increases to 7.7% (95%CI: 1.034, 1.121), *p* < 0.001, where the statistical significance is increased. For men, the risk increases to 6.4% at 2 to 3 days lag, and reaches the maximum of 7.7% at 7 days lag (95%CI: 1.021, 1.136), which passes the significance test of *p* < 0.05. While for women, it is still the same day that has the greatest impact risk and no lagged effect is found. In conclusion, the synergistic effect of air temperature and ozone has the highest risk for women and no lag effect is found, while for men there is a lag effect of 2 to 3 days and 7 days. The total number shows three high risk values on the same day, 3 days, and 9 days, respectively.

**Table 4:** The relative risk and 95% confidence interval (95%CI) of the effect of temperature increases of 1°C with different lag days on the total number of circulatory system deaths and gender in Shijiazhuang.

T (°C)	Total number	Men	Women
T	1.068 (1.025, 1.114)*	1.046 (0.988, 1.109)	1.093 (1.029, 1.162)*
T.1	1.061 (1.020, 1.103)*	1.045 (0.990, 1.102)	1.079 (1.020, 1.142)*
T.2	1.065 (1.025, 1.106)*	1.064 (1.010, 1.121)*	1.066 (1.009, 1.127)*
T.3	1.069 (1.029, 1.111)**	1.064 (1.009, 1.122)*	1.075 (1.016, 1.13)*
T.4	1.065 (1.025, 1.107)*	1.061 (1.006, 1.118)*	1.071 (1.013, 1.132)*
T.5	1.067 (1.027, 1.108)**	1.066 (1.011, 1.123)*	1.068 (1.010, 1.128)*
T.6	1.066 (1.026, 1.107)**	1.072 (1.017, 1.129)*	1.059 (1.003, 1.119)*
T.7	1.071 (1.030, 1.113)**	1.077 (1.021, 1.136)*	1.065 (1.006, 1.126)*
T.8	1.074 (1.033, 1.11)**	1.075 (1.018, 1.135)*	1.074 (1.014, 1.137)*
T.9	1.077 (1.034, 1.121)**	1.070 (1.013, 1.131)*	1.084 (1.022, 1.149)*

*p < 0.05; **p < 0.001.

## 4. Discussion

It is found that the effects of daily mean temperature and ozone pollution on the number of deaths from circulatory diseases in summer are nonlinear. The threshold of daily mean temperature is 27.5°C, and the threshold of ozone pollution is 100 μg/m^3^. High temperatures in summer increase the risk of death from circulatory diseases (14, 15). In terms of the temperature threshold index, the local comfortable temperature is used as a reference in many Chinese cities (16, 17) to obtain the temperature threshold that has an impact on the number of deaths from cardiovascular and cerebrovascular diseases. For example, the temperature in Chengdu, Harbin, Changsha, and Guangzhou is 22.2°C, 20.6°C, 25.1°C, and 26.5°C, respectively. In Shanghai (18), the median daily mean temperature of 18.2°C is taken as the reference, and the risk effect of high temperatures of 30.1°C (95th percentile of temperature) on stroke can reach 26%. In Hong Kong (19), the 75th percentile of 27.8°C is used as the control, and the mortality risk of cardiovascular and cerebrovascular accidents is 9% (95%CI: 1.006, 1.125) when the temperature is higher than 31.5°C (99th percentile). Giang PN et al. (20) showed that when the average temperature in Vietnam is higher than 26°C, the risk of admission for cardiovascular and cerebrovascular diseases increase with the increase of temperature. In this paper, aiming at the influence of summer temperature on circulatory diseases, the threshold of 27.5°C is higher than the annual comfortable temperature, but it is equivalent to the 55th percentile for summer. The death risk of circulatory system diseases caused by high temperatures is mainly related to heat stimulation of the nervous regulation of the circulatory system, increased sweating, blood viscosity, blood vessel dilation, accelerated blood circulation, tachycardia, blood pressure changes, and internal blood insufficiency (21).

When O_3_ concentration is higher than 100 μg/m^3^, the risk of daily deaths from circulatory diseases in Shijiazhuang increased by 3.7% with every increase of 10 μg/m^3^ in O_3_ concentration, and the risk is statistically significant (*p* < 0.05). Gu et al. (22) studied the exposure-response relationship between ozone and the number of emergency patients with cardiovascular and cerebrovascular diseases in Ningbo, and showed that when ozone concentration increased by 10 μg/m^3^ in the warm season, the number of emergency patients with cardiovascular and cerebrovascular diseases increased by 1.17%. Tao et al. (23) studied the acute effects of ozone pollution in the Pearl River Delta, and the total mortality rate increased by 0.81% when ozone concentration increased by 10 μg/m^3^, which is consistent with the results of this study. Dong et al. (24) conducted a meta-analysis of short-term ozone exposure and mortality risk in a Chinese population, and demonstrated that the rise of atmospheric ozone concentration would lead to an increase in non-accidental total mortality, cardiovascular system disease mortality, and respiratory system disease mortality. However, Hu et al. (25) studied the relationship between atmospheric ozone concentration and residents’ first aid in Shijiazhuang city from 2013 to 2015 and found that, when ozone concentration increased by 10 μg/m^3^, the number of residents requiring first aid for respiratory diseases increased by 1.21%, but there was no significant change in the number of residents requiring first aid for circulatory diseases. This may be related to seasonal differences and whether to adjust the confounding effect of PM_2.5_ pollutant (22, 26). This study mainly focuses on summer and adjusts the confounding effect of PM_2.5_. Ozone has a strong oxidizing ability, and short-term exposure to ozone causes the increase of systemic oxidative stress, which is related to human platelet activation and blood pressure increase, thus affecting cardiovascular health (27, 28).

The synergistic effect of temperature and ozone on population health is less studied. The North China Plain is a region with high temperatures and ozone concentrations in the summer (11). It is found that, when O_3_ ≥ 100 μg/m^3^ and *T* ≥ 27.5°C, the risk of death in the circulatory system is the highest 6.8% (95%CI:1.025, 1.114), with it growing with every 1°C increase in temperature. When O_3_ < 100 μg/m^3^ and *T* ≥ 27.5°C, the risk of circulatory death is still 6.8% (95%CI:0.929, 1.228), but is not statistically significant. When *T* < 27.5°C, no risk relationship is found whether ozone concentration is greater than 100 μg/m^3^ or not. It shows that high temperatures mainly affect the death number of circulatory system diseases in summer, and high temperatures and high ozone pollution have a synergistic enhancement effect. The study by Zhang et al. (16) showed that the interaction between air temperature and pollutants had a very complex relationship on the number of deaths from diseases. When high temperatures and high ozone concentration co-existed, there was a synergistic strengthening effect on the number of deaths from respiratory and cardiovascular diseases, which was consistent with the results of this study. Zhang (29) studied the interaction effect between air temperature and PM_2.5_ pollutant in Beijing and showed that when the air temperature was higher than 24°C, the risk of death from cardiovascular and cerebrovascular diseases caused by air temperature and PM_2.5_ together reached 3.97%, which increased the risk of death from circulatory diseases in a high temperature and high pollution environment. Ren C (30) studied the short-term effects of air temperature and ozone on the total mortality in 60 communities in the eastern United States and pointed out that high temperatures could regulate the risk of ozone death, with certain regional differences.

In the study of the lag effect and gender difference, this study finds that under a high temperature and high ozone concentration environment, there is a 3-day lag effect on the total number of deaths on men from circulatory system diseases, but there is a lag effect on women. Zhang et al. (16) performed a single ozone lag analysis and found that the risk of death from cardiovascular and cerebrovascular diseases increased by 0.66% (95%CI: 0.42, 0.90) for every increase of ozone concentration of 10 μg/m^3^ after 1 day’s lag. Cao et al. (31) analyzed the impact of high temperatures and heat waves on death from cardiovascular and cerebrovascular diseases in Jinan and found that there was a lag of 1 to 2 days. Gu et al. (22) analyzed the influence of ozone concentration on cardiovascular and cerebrovascular diseases by using the reception data of emergency vehicles in Ningbo and found that when ozone concentration increased by 10 μg/m^3^, the excess risk of the number of emergency patients for cardiovascular and cerebrovascular diseases was greater for men than women, and there was no lag effect. There are some similarities and differences between the above studies and the results of this study. In many studies, there is a lag analysis for high temperature, and there is also a lag analysis for the impact of pollutants, but the analysis of the synergistic lag of temperature and ozone is rare. Theoretically, there is a lag effect on circulatory disease from high temperatures, and there is also a lag effect from ozone. The high temperature and high ozone environment in summer enhances the risk of circulatory death, and the lag results obtained in this study are reliable.

In this study, Shijiazhuang, a representative city in northern China with frequent high temperatures in summer and serious ozone pollution, is selected. Disease data are circulatory system disease death data, and the selected cities and health conditions are more representative than outpatient case data. This study analyzes the single factor influence relationship, threshold index, synergistic effect, and lag effect of temperature and ozone, which is more comprehensive than previous analysis. It reflects the relationship based on the impact of temperature and ozone pollution on the number of deaths from circulatory system diseases, which is a common issue, but there may be certain limitations in individual exposure.

## 5. Conclusion

In this paper, daily circulatory system disease death data, meteorological data, and O_3_ and PM_2.5_ concentration pollution data in Shijiazhuang city from June to August in the summers of 2013 to 2016 were used to evaluate the impact of high temperatures and short-term exposure to O_3_ on the number of deaths from circulatory system diseases after controlling the confounding effect. It was found that high temperatures and O_3_ pollution had a synergistic effect on circulatory system diseases. It provides evidence for strengthening the prevention and scientific management of circulatory system diseases under high temperatures and in high ozone environments.

## Data availability statement

The original contributions presented in the study are included in the article/supplementary material, further inquiries can be directed to the corresponding author.

## Author contributions

GQF: Formal analysis, Methodology, Writing – original draft. HMC: Data curation, Writing – original draft. QL: Writing – review & editing. HYL: Validation, Writing – original draft. XHZ: Data curation, Writing – original draft. XSZ: Visualization, Writing – original draft.
